# Overlap-Based Cell Tracker

**DOI:** 10.6028/jres.115.034

**Published:** 2010-12-01

**Authors:** Joe Chalfoun, Antonio Cardone, Alden A. Dima, Daniel P. Allen, Michael W. Halter

**Affiliations:** Software and Systems Division; Biochemical Science Division, National Institute of Standards and Technology, Gaithersburg, MD 20899

**Keywords:** cell motility, live-cell imaging, overlap-based cell tracking, time-lapse cell imaging

## Abstract

In order to facilitate the extraction of quantitative data from live cell image sets, automated image analysis methods are needed. This paper presents an introduction to the general principle of an overlap cell tracking software developed by the National Institute of Standards and Technology (NIST). This cell tracker has the ability to track cells across a set of time lapse images acquired at high rates based on the amount of overlap between cellular regions in consecutive frames. It is designed to be highly flexible, requires little user parameterization, and has a fast execution time.

## 1. Introduction

Automated microscopy has facilitated the large scale acquisition of live cell image data [Sig06, Gor07, Dav07, and Bah05]. In the case of low magnification imaging in transmission mode, the migration, morphology, and lineage development of large numbers of single cells in culture can be monitored. However, obtaining quantitative data related to single cell behavior requires image analysis methods that can accurately segment and track cells. When fluorescence protein gene reporters are used, the activity of specific genes can be related to phenotypic changes at a single cell level. The analysis of living, single cells also provides information on the variability that exists within homogeneous cell populations [Ras05 and Si206]. Furthermore, multiple fluorescence protein reporters transfected into single cells can be used to understand the sequence of transcriptional changes that occurs in response to perturbations. In order to facilitate the extraction of quantitative data from live cell image sets, automated image analysis methods are needed.

The diversity of both cell imaging techniques and the cell lines used in biological research is enormous making the task of developing reliable segmentation and cell tracking algorithms even harder. Many popular cell tracking techniques are based on complex probabilistic models. In [Bah05] Gaussian probability density functions are used to characterize the selected tracking criteria. In [Mar06] cells are tracked by fitting their tracks to a persistent random walk model based on mean square displacement. In [Lia08] the final cell trajectories and lineages are established based on the entire tracking history by using the interacting multiple models (IMM) filter [Gen06]. In [Kha05], a Markov Chain Monte Carlo based particle filter is used to initially detect the position of the targets and then a Rao-Blackwellized particle filter is applied. An important class of tracking techniques consists of level set methods [Bes00, Man02, and Shi05]. They produce fairly accurate tracking results but are difficult to implement and computationally expensive. The tracking techniques proposed in [Dor02, Ray02, Zim02] are commonly referred to as active contour or snake techniques. In general they do not consider all possible tracking candidates in the frame, but focus on the candidates corresponding to a predefined model (e.g., located around a reference initial position). Finally, tracking techniques based on mean-shift algorithms provide a fast solution, but often do not provide accurate information about object contours [Col03, Com03, Deb05]. Many available techniques are computationally expensive and have a large number of parameters to adjust for every track. We propose a new technique that can produce accurate tracking with a small set of adjustable parameters in situations where cell movement between consecutive frames is limited so that there is typically some cell pixel overlap between frames.

**Fig. 1:**
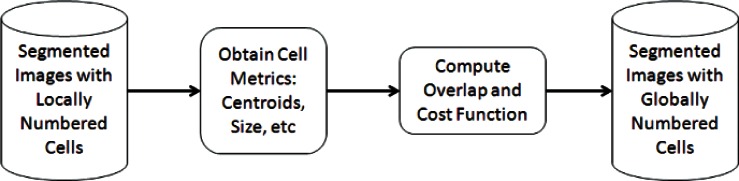
Core algorithm

Our experience shows that when acquiring time-lapse images at intervals ranging from 5 min to 15 min, the movement of cultured mammalian cells between two consecutive frames will be relatively small. This means that between consecutive frames a typical cell will occupy nearly the same position. In order to effectively analyze large volumes of data (> 10 000 images) an automated process requiring very little manual intervention and involving a simple and meaningful set of parameters is needed. The overlap-based cell tracking software developed by NIST was designed with this goal in mind. It tracks cells across a set of time lapse images based on the amount of overlap between cellular regions in consecutive frames. It is designed to be highly flexible and suitable for use in a wide range of applications, requires little user interaction during the tracking process, and has a fast execution time. Though it requires that the change in a cell’s location from one frame to the next be relatively small to work reliably, acquiring images at 5 min to 15 min intervals is feasible with standard automated live cell imaging systems and provides image data that is suitable for an overlap-based algorithm. The core tracking algorithm is shown in Fig. 1.

In this paper, a general formulation of the motion tracking problem will be given, followed by a brief description of the input data and of the tracking criteria employed. Some instances of application of the tracking software will be presented to further illustrate its capabilities. We will conclude with a brief summary of our results.

## 2. Problem Statement

**Fig. 2:**
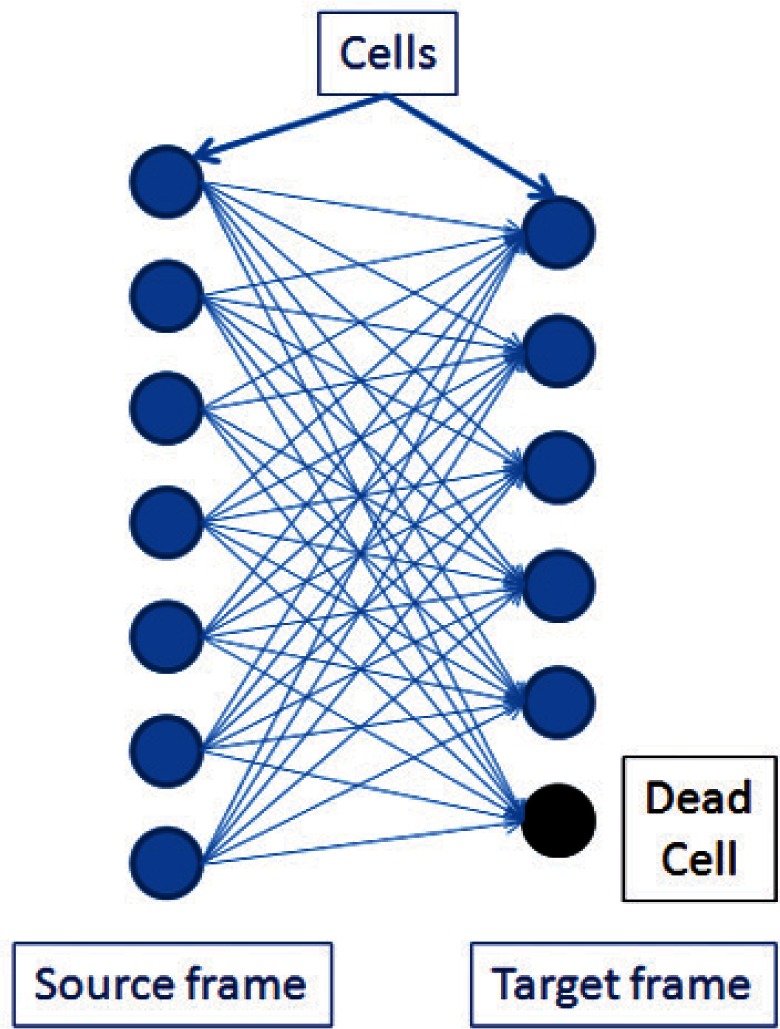
Possible combinatorial tracking between two consecutive frames.

Cellular tracking techniques are used to obtain motion and life cycle behavior information about cells by following the cells of interest through multiple, time sequential images. The cell tracking problem can be defined as: given a cell A from a current (source) image, identify the corresponding cell B, if any, in the subsequent (target) image. If cell A is tracked to B, then the two cells are the same cell at successive moments in time. This process involves examining all possible combinatorial mappings of the cells in a source image to the cells in the target image (Fig. 2) and finding the optimal mapping. The process is then repeated using the target image as the source image and the next image in the set as the target image until the entire set of images has been traversed. The image to image mappings are then chained together to form a complete life-cycle track of every individual cell in the image set.

**Fig. 3:**
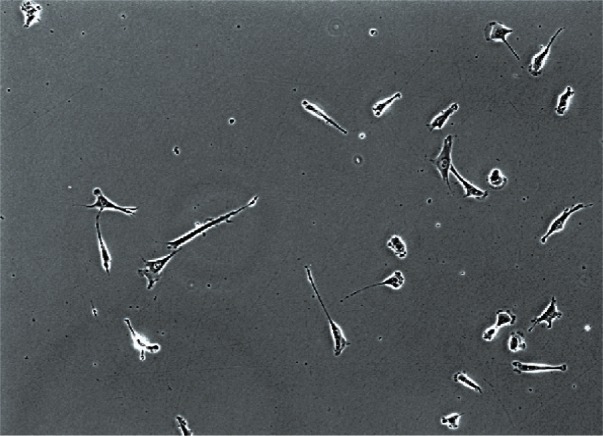
Example of a phase contrast microscopy image.

Many different types of imagery can be obtained with modern cellular microscopy instruments—in our case we will be working with phase contrast images of NIH-3T3 fibroblasts, shown in Fig. 3 below.

## 3. Image Data and Preliminary Definitions

The input of the tracking algorithm is a series of segmented images (masks) derived from the raw microscopy data. The masks identify the individual pixels in an image that correspond to a cellular region and are generated from the raw phase contrast microscope images using automated image segmentation. Many segmentation techniques exist in the literature; some are general purpose and others are specific to a cell line and/or image acquisition parameters. The specifics of the segmentation algorithm used in this project will not be addressed here and in general the NIST cell tracking algorithm can be used with any segmentation algorithm. It is important to note however that the reliability of the tracking outcome is highly dependent on the accuracy of the segmentation.

**Fig. 4:**
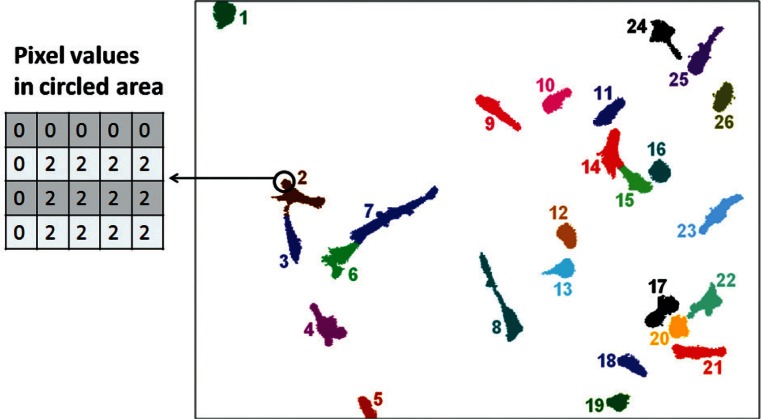
Segmented image mask for the example image in Fig. 3.

The notation used to refer to a segmented image or mask is *I_k_*, with *k* = 1,2, …, *N*, *I_k_* is the *k*th image in the set and *N* is the total number of images in the set. The segmentation process sets the value of all background pixels in the mask to zero. It sets the value of all pixels segmented into a cellular region to a positive integer value called the cell number (Fig. 4). The cell numbers are assigned to each segmented region starting at 1 and continuing incrementally until all segmented regions have been labeled. The regions are numbered in the order in which the cells are encountered. The notation used to represent a given pixel at a location in the image is *p*(*x*, *y*), where:

(1) $$p(x,y)=\{\begin{array}{cc} 0 & p(x,y)\in background\\ i>0 & p(x,y)\in c_{i}^{k} \end{array}$$

The notation $c_{i}^{k}$ is used to identify cell number *i* from the *k*th image. *i* = 1,2,…, *M_k_*. *M_k_* represents the total number of cells that are present in the *k*th image. For visual clarity, each number is also represented by a unique color when plotted. Figure 4 shows the segmented image generated from the phase contrast image in Fig. 3.

## 4. The Overlap-Based Tracking Concept

The NIST cell tracking algorithm computes a cost for each possible cell-to-cell mapping based on some simple tracking criteria. The cost value represents a measure of the probability that cell $c_{i}^{k}$ from image *I_k_* should be tracked to cell $c_{j}^{\left(k+1\right)}$ in the subsequent image. The cost function has been defined in such a way that the higher the cost value is, the lower the probability that the two cells should be identified as being the same cell across frames. A general definition of the cost function between a pair of cells from two different images is given as follows:

(2) $$d\left(c_{i}^{k},c_{j}^{k+1}\right)=f(\text{tracking}\;\text{criteria})$$

**Fig. 5:**
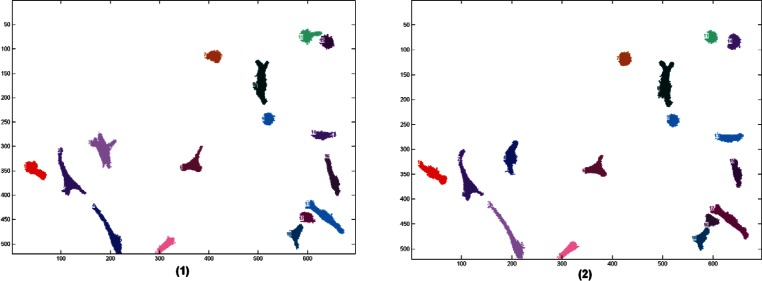
Image 1 and Image 2—two consecutive segmented images.

**Fig. 6:**
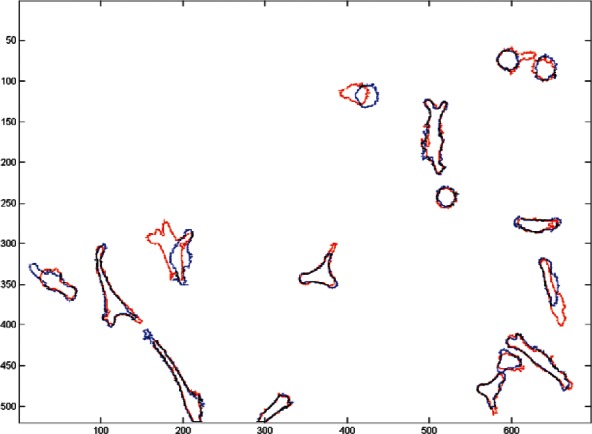
Image 1 (red outline) superimposed on Image 2 (blue outline).

Before describing in detail the tracking criteria used in this paper, consider the two consecutive segmented phase-contrast images shown in Fig. 5 below. Note, that individual cells do not significantly change their position between consecutive frames. This is more easily seen in Fig. 6 where the images are superimposed. This suggests that the number of common pixels (the overlap) between a pair of cells can be used as the principal measure of cost. If a pair of cells shares a large number of overlapping pixels, then these two cells are most likely the same cell in different images. If more than two cells overlap we will need to employ additional criteria to further refine the cost. It is important to note that for this technique to work reliably the images must be acquired at a sufficiently high rate to minimize cell movement between successive frames. If the images are too far apart in time the cells may migrate great distances across the image window and will exhibit little or no overlap. At low acquisition rates cell motion may appear so chaotic that even a human observer will find it difficult to identify them correctly. The acquisition rate used for the NIST 3T3 cells tracked in this paper is typical for this type of cell line.

The cost function uses the following criteria for computing the cost of a mapping:

1. The amount of overlap between source and target cells.
2. The Euclidean distance (offset) between the centroids of the source and target cells.
3. The difference in size between the source and target cells.

The metrics used for quantifying these criteria are normalized between 0 and 1. A value of zero denotes a perfect match between a pair of cells: all pixels overlap, the centroids are in the same location and cells have the same size. The cost function is defined as a sum of the individual metrics, each representing a tracking criterion. Hence, lower values of the cost function indicate a higher probability that the source and target cells are the same cell. This mathematical representation carries desirable properties such as differentiability and the ease of including additional tracking criteria by adding new terms. Since the terms of the summation were defined in such a way that they are independent, they can be modified as needed without affecting the remaining terms.

A more complete mathematical statement of the cost function is:

$$d\left(c_{i}^{k},c_{j}^{k+1}\right)=w_{o}\times O\left(c_{i}^{k},c_{j}^{k+1}\right)+w_{c}\times \delta_{c}\left(c_{i}^{k},c_{j}^{k+1}\right)+w_{s}\times \delta_{s}\left(c_{i}^{k},c_{j}^{k+1}\right)$$

where:

*w*_o_ = the weight of the overlap term,
*O* = an overlap metric,
*w_c_* = the weight of the centroid offset term,
δ*_c_* = a centroid offset metric,
*w_s_* = the weight of the cell size term, and
δ*_s_* = a cell size metric.

The weights are provided for flexibility and allow the basic algorithm to be tailored for use with different cell lines and image acquisition conditions. For example if the image acquisition rate were high and cells overlap greatly between two consecutive frames then *w*_o_ should be set to a high value. If the size of the cells changes very little between two consecutive frames then a larger weight can be given for the size term. The weights used in the examples presented in this paper are:

$$w_{o}=2,w_{c}=1,\text{and}\;w_{s}=0.5.$$

### 4.1 Pathological Filtering

Some source/target pairs are so obviously undesirable that they are filtered prior to applying the cost function. Specifically, if the source and target cells have no pixels in common *and* the distance (in pixels) between their centroids is greater than a user defined threshold value, then the mapping is assigned an arbitrarily high cost (MAX_COST) to ensure that it will never be chosen. For example, a cell in the upper right corner should not be tracked to a cell in the lower left corner (cells don’t jump that much between consecutive frames). By definition mappings with a cost of MAX_COST are invalid. This filtering is derived from common sense and experience with cell biology and cell morphology.

### 4.2 The Overlap Metric

The overlap metric for a source/target pair is a measure of the number of pixels the two cells have in common between two consecutive frames. It is computed using the formula:

$$O(c_{i}^{k},c_{j}^{k+1})=1-\left[\frac{n_{o}(c_{i}^{k},c_{j}^{k+1})}{2}\left(\frac{1}{s_{i}^{k}}+\frac{1}{s_{j}^{k+1}}\right)\right]$$

where:

$s_{i}^{k}$ = the size in pixels of the source cell,
$s_{j}^{\left(k+1\right)}$ = the size in pixels of the target cell, and
$n_{o}(c_{i}^{k},c_{j}^{\left(k+1\right)})$ = the number of pixels the two cells have in common.

### 4.3 The Centroid Metric

The centroid metric is a measure of the Euclidean distance between the centroids of the source and target cells between two consecutive frames. Let the width and height (in pixels) of a frame be represented by the symbols *I*_width_ and *I*_height_ and denote the centroid coordinates (in pixels) of cell *i* in frame *k* by the symbols $\left(X_{i}^{k},Y_{i}^{k}\right)$. The centroid metric for a source/target pair is computed as:

$$\delta_{c}(c_{j}^{k},c_{j}^{k+1})=\frac{\sqrt{{\left(X_{i}^{k}-X_{j}^{k+1}\right)}^{2}+{\left(Y_{i}^{k}-Y_{j}^{k+1}\right)}^{2}}}{\sqrt{I_{\text{height}}^{2}+I_{\text{width}}^{2}}}.$$

### 4.4 The Size Metric

The size metric is a measure of the relative difference in the sizes of the source and target cells in two consecutive frames. It is computed as:

$$\delta_{s}(c_{i}^{k},c_{j}^{k+1})=\frac{\left|s_{i}^{k}-s_{j}^{k+1}\right|}{\max(s_{i}^{k},s_{j}^{k+1})}.$$

### 4.5 Tracking Solution

Once the individual cell mappings between consecutive frames have been computed, the frame-to-frame mappings are combined to produce a complete life cycle track of all the cells in the set of images. The sequentially assigned cell numbers given by the segmentation process for the cells in each frame are replaced with uniquely numbered track numbers that identify the movement of each cell in time across the entire set of images. Therefore a unique track number *t_n_* will be associated to each uniquely identified cell, *n* = 1,2, …, *T* where *T* represents the total number of unique cells found in the image set. The pixels in the images are relabeled to reflect the new track numbers such that when a pair of cells has been assigned with a tracking number the pixels from all images that belong to a given cell will all have the same value.

$$\begin{array}{c} \text{if}\;c_{i}^{k}\overset{t_{n}}{↔}c_{j}^{k+1}\Rightarrow c_{i}^{k}=t_{n}=c_{j}^{k+1}\\ \Rightarrow \forall x,y/p(x,y)\in (c_{i}^{k}\cup c_{j}^{k+1}),p(x,y)=t_{n}. \end{array}$$

**Fig. 7:**
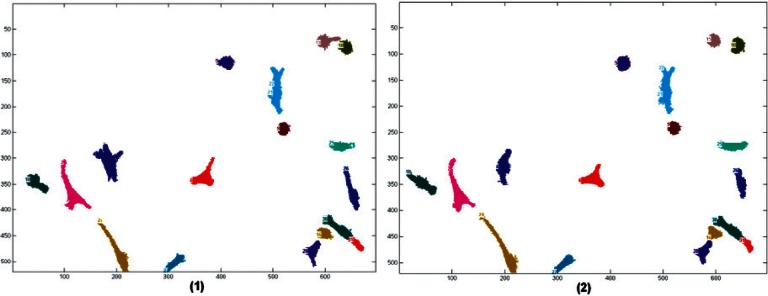
Two consecutive tracked images. The cells that were identified as being the same were given the same number and color in both images.

In Fig. 5, in each segmented image, the cells were numbered randomly from 1 to max. When these cells are given a global number, they will carry the same number thru time. Figure 7 shows that this is also reflected by the colors of the cells, the same cell will have the same color throughout the images.

## 5. Results and Outputs

**Fig. 8:**
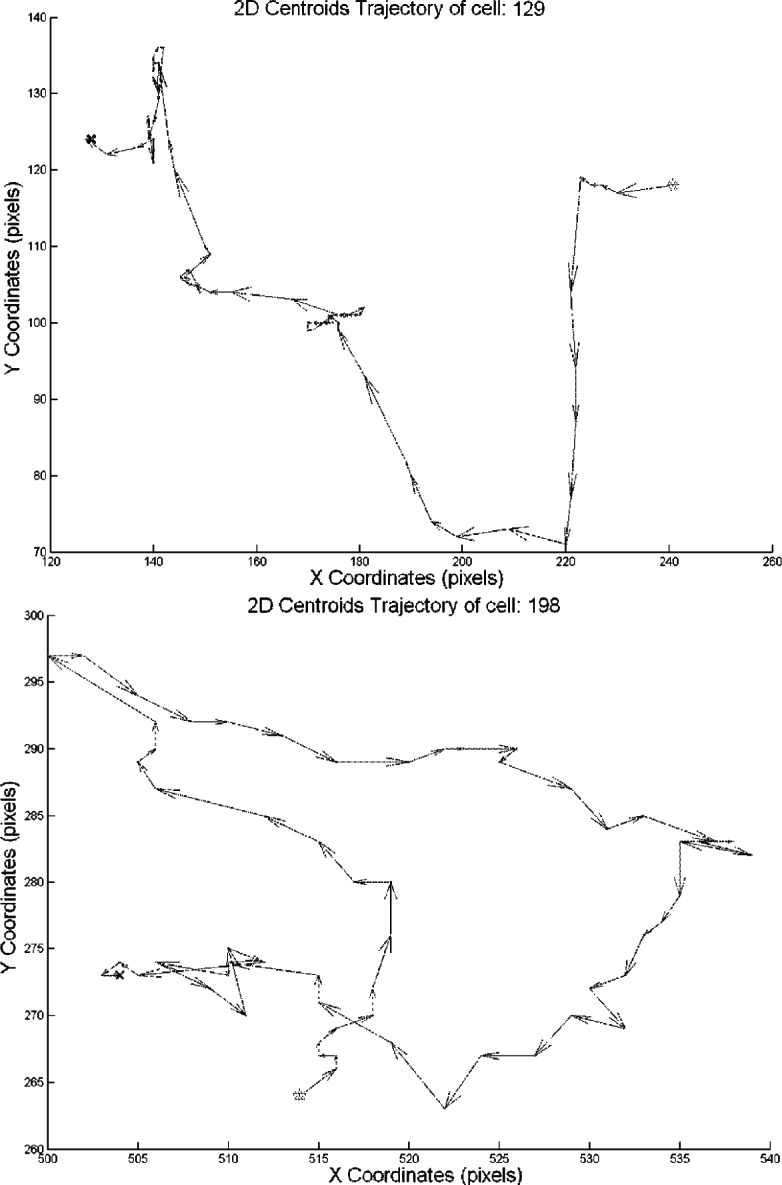
2D cell centroid trajectories. Each arrow in the image represents the direction and the distance traveled by the cell between two consecutive frames. There is 15 min interval between each frame.

**Fig. 9:**
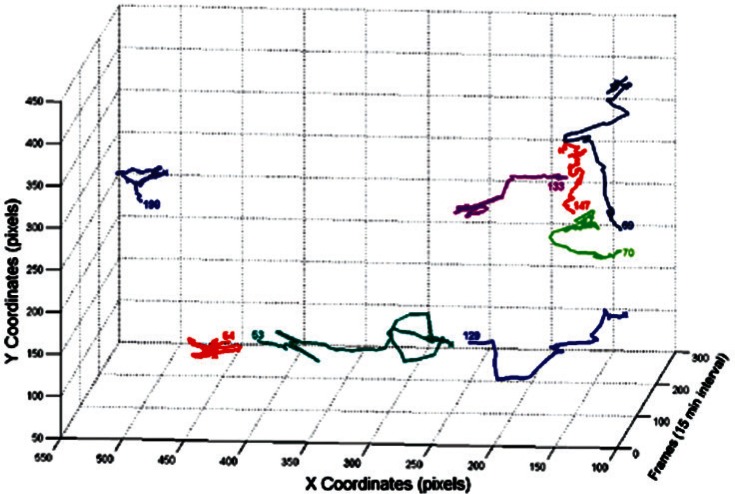
3D cell centroid trajectories for some cells.

**Fig. 10:**
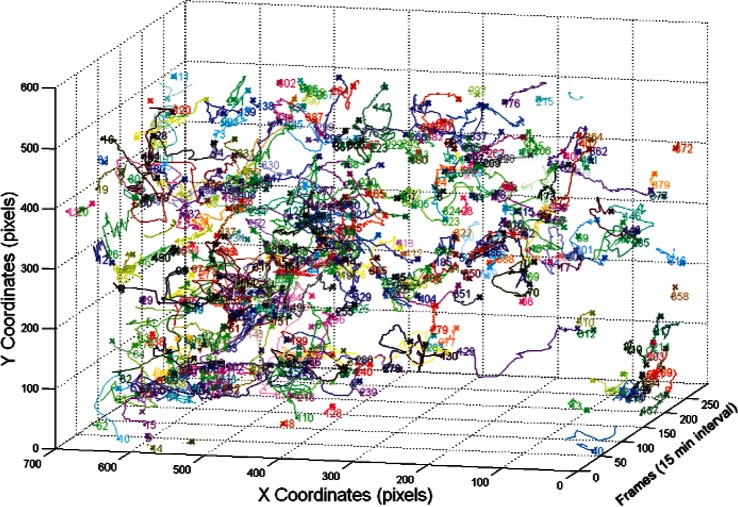
3D cell centroid trajectories for all cells.

After applying the cell tracker on the segmented images, the results are documented and saved in the cell tracker output folder as matrices. This enables fast access to the output when needed. Figures 8, 9 and 10 show the centroid trajectories of the cells in 2D and 3D. This will help to determine the traveling rate of cells.

## 6. Conclusion

An overlap cell tracking software developed by NIST was described. This cell tracker has the ability to track cells across a set of time lapse images acquired at high rates based primarily on the amount of overlap between cellular regions in consecutive frames. It was designed to be highly flexible, requires little user parameterization, and has a fast execution time.

Future enhancements are planned for the cell tracker. The ability to detect mitosis (when a source cell divides into two new cells) will be added along with capability of detecting colliding cells and giving a feedback to segmentation when such behavior occurs. A cell shape metric will be used to add a shape weight to the cost function. This metric was not needed for tracking the 3T3 fibroblasts as they typically change shape rapidly between consecutive frames. However, a shape-based metric is in general needed to improve the tracking of cell lines or other objects that are more morphologically stable and it should increase the cell tracker’s suitability for use in a wider range of applications.

The average computation time for tracking 500 cells in our set of 252 images (520 × 696 pixels) on a single core Pentium 3.4 GHz 3 GB RAM is 47 s. This translates to an average speed of 5.36 frames/s.
